# Novel Heparin Receptor Transmembrane Protein 184a Regulates Angiogenesis in the Adult Zebrafish Caudal Fin

**DOI:** 10.3389/fphys.2017.00671

**Published:** 2017-09-07

**Authors:** Sara Lynn N. Farwell, Kimberly G. Reylander, M. Kathryn Iovine, Linda J. Lowe-Krentz

**Affiliations:** Department of Biological Sciences, Lehigh University Bethlehem, PA, United States

**Keywords:** angiogenesis, endothelial cells, cell proliferation, heparin, zebrafish, regeneration

## Abstract

Transmembrane protein 184A (TMEM184A) was recently identified as the heparin receptor in vascular cells. Heparin binds specifically to TMEM184A and induces anti-proliferative signaling *in vitro*. Though it is highly conserved, the physiological function of TMEM184A remains unknown. The objective of this study was to investigate the expression and effects on vascular regeneration of TMEM184A using the adult zebrafish regenerating caudal fin as an *in vivo* model. Here, we show that Tmem184a is expressed in vascular endothelial cells (ECs) of mature and regenerating zebrafish fins. Transient morpholino (MO)-mediated knockdown of Tmem184a using two validated MOs results in tangled regenerating vessels that do not grow outward and limit normal overall fin regeneration. A significant increase in EC proliferation is observed. Consistent with *in vitro* work with tissue culture vascular cells, heparin has the opposite effect and decreases EC proliferation which also hinders overall fin regeneration. Collectively, our study suggests that Tmem184a is a novel regulator of angiogenesis.

## Introduction

Heparan sulfates (HS) are long chains of polysaccharides covalently attached to a class of proteins called proteoglycans (HSPGs). Though HSPGs are expressed ubiquitously, their functions are tissue specific and depend on certain properties of the glycosaminoglycan chains (i.e., number of chains, length, charge) and the core protein. These chains have binding sites for several different types of proteins, and these interactions are a critical part of the regulation of many biological processes. One notable example is angiogenesis, the development of new vasculature from existing vessels (Chiodelli et al., 2015). Angiogenesis is required during early development, continues throughout life to accommodate new tissue growth and occurs in response to injury. There are several key cellular behaviors underlying angiogenesis: endothelial cell (EC) differentiation and proliferation, EC migration toward angiogenic signals, modulation of permeability to allow perfusion into the growing tissue, and EC survival once in a new environment. HSPGs expressed on ECs coordinate dynamic interactions between several growth factors and their receptors which ensure proper angiogenesis. For example, HSPGs bind to vascular endothelial growth factor (VEGF) and facilitate interactions with its receptor, VEGFR2, and other co-factors (Couchman et al., 2016). Several members of the fibroblast growth factor family and their receptors also interact with HSPGs and other cofactors, such as integrins as part of the pro-angiogenic process (Chiodelli et al., 2015). Other interactions between HSPGs and receptors that signal differentiation (e.g., Notch/delta) are also important for vascular outgrowth (Roca and Adams, 2007). Though these types of interactions have been documented in the vasculature, knowledge of the mechanism of HSPG involvement during angiogenesis remains incomplete.

Transmembrane protein 184A (TMEM184A) was recently identified as the heparin receptor in vascular ECs and smooth muscle cells (VSMCs) (Farwell et al., 2016; Pugh et al., 2016). Though TMEM184A is highly conserved, its physiological function remains unknown. *In vitro*, TMEM184A is required for potent anti-proliferative and anti-inflammatory signaling cascades induced by heparin (Gilotti et al., 2014; Farwell et al., 2016; Pugh et al., 2016). Exogenous heparin administration has been used in clinical and research settings to identify and characterize novel heparin binding proteins (Sarrazin et al., 2011; Schultz et al., 2017). Heparin binds specifically to TMEM184A and decreases growth-factor induced MAPK signaling and proliferation in VSMCs (Pugh et al., 2016). In ECs, TMEM184A is required for heparin-induced expression of dual specificity phosphatase-1 (DUSP1) and subsequent downregulation of MAPK signaling (Farwell et al., 2016). *In vivo*, it is probable that HS (rather than heparin) interacts normally with TMEM184A. We hypothesized that TMEM184A functions specifically in the vasculature *in vivo* to modulate vascular cell proliferation and other behaviors. To begin to elucidate the *in vivo* function of TMEM184A, we employed the adult zebrafish regenerating caudal fin as a model for angiogenesis/wound healing.

The regenerating zebrafish caudal fin has been utilized as an *in vivo* model for angiogenesis research for over a decade (Chávez et al., 2016). Major players involved in the mechanism of angiogenesis (i.e., growth factors and their receptors) are conserved in zebrafish and function similarly (Poss et al., 2000; Bayliss et al., 2006). Zebrafish also express several HSPGs including syndecans which have been shown to promote angiogenesis by signaling through the Vegfr2 complex (Chen et al., 2004, 2005; Gorsi et al., 2014; Venero Galanternik et al., 2015). The ease of visualizing and quantitating vascular regeneration in the caudal fin has been demonstrated (Huang et al., 2003; Santoro, 2014; Hlushchuk et al., 2016) in part due to the availability of various transgenic reporter lines, such as *Tg(Fli1:EGFP)* which expresses EGFP specifically in ECs. Upon amputation of the caudal fin, a plexus is formed and new vessels develop over several days. The new ECs do not arise from progenitor stem cells but rather sprout from the existing vessels (Huang et al., 2003; De Smet et al., 2014). Thus, vascular regeneration in this model system represents true angiogenesis. Interestingly, in the regenerates, arterial cells develop from venous precursors implying a very specific reorganization that may be crucial for regenerative repair (Kametani et al., 2015). This process of reorganization is necessarily coupled to the activities of angiogenesis noted above for development of mature vessels in the zebrafish fin. Similarly, reorganization to specific tissue requirements is likely true in other situations where angiogenesis occurs.

Here we report the *in vivo* expression and function for heparin receptor Tmem184a. Tmem184a is expressed specifically in zebrafish vasculature. Knockdown of Tmem184a using two different morpholinos (MOs) results in excess EC proliferation and disorganized neovascularization with hindered outgrowth. Heparin injection into regenerating vasculature decreases EC proliferation and affects overall fin regeneration. These results are remarkably consistent with our previous *in vitro* work. Together, these findings suggest that Tmem184a is a modulator of angiogenesis. Our study emphasizes the regenerating zebrafish fin as a strong method to study vascular regeneration and highlights the existence of a novel player in the mechanism of angiogenesis.

## Materials and methods

### Statement on the ethical treatment of animals or zebrafish care and lines/housing and husbandry

This study was performed strictly according to the recommendations in the Guide for the care and Use of Laboratory Animals of the National Institutes of Health. Lehigh's Institutional Animal Care and Use Committee (IACUC) approved the protocols performed in the manuscript (Protocol # 172 approved initially 11/17/2014 and renewed 11/15/2016, 11/28/2016). Lehigh University's Animal Welfare Assurance Number is A-3877-01. All experiments were performed to minimize pain and discomfort. The zebrafish (*Danio rerio*) C32 strain described previously (Rawls et al., 2003) was used as the animal model for this study. The fish were housed in a re-circulating system built by Aquatic Habitats (now Pentair). 1.5 L tanks (up to 6 fish/tank) were used. The fish room was set to a 14:10 light:dark cycle and the room temperature was kept between 27 and 29°C. Water quality was monitored automatically and dosed to maintain conductivity (400–600 μs) and pH (6.95–7.30). A 10% water change was performed daily. A biofilter was used to control and maintain nitrogen levels. System water (i.e., recirculating water) was filtered sequentially through pad filters, bag filters, and a carbon canister before circulating over ultraviolet lights for sterilization. Fish were fed three times daily, once with brine shrimp (hatched from INVE artemia cysts) and twice with flake food (Zebrafish Select Diet, Aquaneering Inc.).

### Zebrafish strains and surgical procedures

The *Tg(Fli1:EGFP)* transgenic line (as described in Lawson and Weinstein, 2002) was used in this study. Both males and females were used. The number of individuals for each experiment is provided in each figure legend. Caudal fin amputations, fin regeneration, and harvesting were done as previously described (Govindan and Iovine, 2014; Banerji et al., 2016). Fish were anesthetized in 0.1% tricaine solution, and the caudal fin was amputated to 50% using a sterile razor blade under a dissecting microscope. Fish were returned to a tank and monitored until full mobility returned within a few minutes. Fin regeneration proceeded for 3 days post amputation (3 dpa) before injection with morpholino (MO) or treatment. Half of the fin (usually the dorsal fin rays) of *Tg(Fli1:EGFP*) fish was injected for microscopy assays (phenotype assessment, regeneration lengths, cell proliferation). The entire fin of wild-type C32 fish was injected with MO or a specific treatment when preparing fin lysates for western blotting. All fins were harvested 1 day post injection, or 4 dpa.

### Morpholino injection

All morpholinos (MOs) were purchased from GeneTools, LLC (Philomath, OR) and dissolved in sterile ddH_2_O. Two non-overlapping MOs targeting Tmem184a were used: one that targeted the translation start site, ATG MO, and one targeting the splice site between pre-mRNA exons 2 and 3, SS MO. A mismatched standard control (Con MO) was also used to control for off target effects. The ATG MO was tagged with lissamine rhodamine, and the SS MO and Con MO were tagged with fluorescein. Sequences of MOs were as follows: ATG MO 5′CTGAGAGTAGTTTCATTCATCCTGA3′; SS MO 5′AAACAGGCACACTCACTGAATGGGC3′; Con MO 5′CCTCTTACCTCAGTTACAATTTATA3′. Microinjection and electroporation procedures were performed as described previously using a Norishinge IM 300 microinjector and visualized under Nikon SMZ 800 with a 1x objective (Banerji et al., 2016). MO-injected fins were evaluated for vascular regeneration phenotype, total and vascular regeneration length, cell proliferation, and protein expression by western blot and immunostaining.

### Confocal microscopy to assess regenerating vascular phenotype

Initial regenerating vascular phenotypes in Con and ATG MO-injected fins were assessed with confocal microscopy. The regenerating portion of each fin was harvested and fixed in 4% paraformaldehyde (PFA) in phosphate buffered saline (1x PBS) for either overnight at 4°C or for 4 h at room temperature. Fins were dehydrated in 100% methanol and stored in −20°C until further use. Fins were rehydrated from methanol to 1x PBS through a series of washes. Fins were mounted into glycerol on a microscope slide and covered with a coverslip. Fins were imaged for EGFP and lissamine-rhodamine with a 10x objective of a Zeiss LSM 880 confocal microscope at room temperature. Higher resolution tiled z-stacks were taken to image the entire fin and to allow us to zoom in to analyze vasculature around the third and fourth fin rays of each side.

### Heparin injection

At 3 dpa, *Tg(Fli1:EGFP)* fish were anesthetized and 100 μg/mL unfractionated heparin from porcine skin (Sigma), 1% phenol red in 1x PBS, or 100 μg/mL chondroitin sulfate (Sigma) was injected into the third and fourth fin rays of the dorsal regenerating caudal fin. The uninjected ventral fin rays served as an internal control. After 24 h (4 dpa), fins were imaged using a Nikon Eclipse 80i microscope with a 4x objective and a Nikon Eclipse E1000M microscope with 1x or 4x objectives. Brightfield and EGFP fluorescence (FITC filter) images were obtained for both injected and uninjected sides of each fin. The fins were then harvested as the MO-injected fins and processed for vascular phenotype, total and vasculature regenerate length, and cell proliferation analyses.

### Total and vascular regeneration length measurement and analysis

Brightfield and regenerating vasculature (EGFP, FITC filter) images obtained with a Nikon Eclipse E1000M microscope and NIS elements software were exported as TIFs. Regenerating vasculature images were superimposed over the corresponding brightfield image. The transparency of the vasculature image was set to 50–75%, and the brightness and contrast were changed so individual fin rays became clear and easily measurable. Total and vascular regeneration length from the amputation plane of the third fin ray of each side (uninjected vs. injected) of each fin was measured using NIH ImageJ (RRID:SCR_003070) as previously reported (Banerji et al., 2016). Briefly, to measure total fin regeneration, a line was drawn from the amputation plane to the distal end of the tissue, as shown in **Figure 4A**, top. To measure vascular outgrowth, a line was drawn from the amputation plane to the tip of fluorescent vasculature, as shown in **Figure 4A**, bottom. The length of each line was measured and recorded. A ratio of the injected to uninjected lengths for each fin was taken and reported as percent similarity (refer to separate section on percent similarity calculations and statistics below).

### Immunoblotting and Tmem184a antibodies

Tmem184a protein knockdown was confirmed by preparing fin lysates for western blotting as previously described (Banerji et al., 2016) with the following modifications. Approximately 8–12 WT MO-injected (Tmem184a ATG MO, Tmem184a SS MO, or control MO) 1dpe/4dpa regenerating fins were pooled and incubated in RIPA buffer (50 mM Tris HCl, 5.0 M NaCl, 0.1% SDS, 0.1% Triton X-100, 0.5% sodium deoxycholate with pH adjusted to 8.0) supplemented with protease inhibitor (Thermo Scientific, HALT™ Protease and Phosphatase Inhibitor Cocktail, 100X catalog #78430) on ice. Harvested fins were homogenized by a tissue homogenizer (Bio-Gen, PRO 200) at high speed 3 times for 5 s with 5 s cooling intervals. Homogenized samples were incubated on ice for 30 min and centrifuged at 250 × g for 15 min at 4°C. The supernatant was removed, and an equal volume of 2 × SDS sample buffer was added. Samples were immediately boiled and stored in −20°C.

Protein expression was evaluated using fluorescent secondary antibody western blotting as described previously (Farwell et al., 2016). All fluorophore-conjugated secondary antibodies used in this study were obtained from Jackson ImmunoResearch, West Grove, PA. Dilutions and catalog numbers are provided for each secondary antibody. Tmem184a was detected by Western blot using a primary antibody specifically against zebrafish Tmem184a (1:2,000, GenScript). An affinity purified polyclonal antibody was generated against a C-terminal peptide (ZF-CTD, CSGLKETINPGDMVQ). The antigenic region for the C-terminal antibody does not overlap with the putative heparin/HS binding region. Competition blots were performed with the appropriate blocking peptide to confirm specificity. Alexa 647 anti-rabbit (1:5,000; 711-605-152) was used to detect anti-ZF-CTD primary antibody. Mouse anti β-actin (1:10,000 Sigma A-5441) was used as a loading control. Alexa 488 anti-mouse (1:10,000; 715-545-150) or TRITC anti-mouse (1:10,000; 715-025-150) were used to detect the primary antibody against actin. Image J was used to measure band intensities as previously described (Banerji et al., 2016). Relative expression was calculated as the ratio of Tmem184a to actin. The graph shown is representative of three experiments and shows the average relative expression ± SEM. Student's *t*-test was used to compare groups.

### Cell proliferation assay and analysis

Cell proliferation was detected by immunostaining for histone-3-phosphate (H3P) as described previously (Banerji et al., 2016). Antibodies used for H3P assays were pre-adsorbed overnight at 4°C with ontogenic fins. Primary and secondary antibodies used for control MO and Tmem184a ATG MO-injected fins were rabbit anti-H3P (1:100, Millipore 06-570) and Alexa 647 anti-rabbit (1:200; 711-605-152). Primary and secondary antibodies used for Tmem184a splice-site MO-injected fins were rabbit anti-H3P, mouse anti-GFP (1:200; Santa Cruz sc-9996), Alexa 647 anti-rabbit, and Cy3 (715-165-150) or TRITC anti-mouse (1:200; 715-025-150), respectively. The third and fourth fin rays of the uninjected and injected sides of each fin were imaged for EGFP and Alexa 647 with a 20x objective of a Nikon C2+ confocal microscope. Z-stacks were taken for each image in NIS Elements software (RRID:SCR_014329). To analyze only H3P-positive cells in Fli1-positive ECs above the amputation plane, H3P was pseudocolored to red for all fins, and EGFP was pseudocolored to green in SS MO-injected fins. Z stacks/images were projected in 3D using the Show Volume View function and alpha blending was applied. Individual H3P/Fli1-positive nuclei were confirmed by zooming in and rotating in several directions and counted without software.

### Immunostaining in transverse cryosections

*Tg(Fli1:EGFP)* ontogenic and regenerating fins were sectioned as previously described (Govindan and Iovine, 2015). Briefly, previously fixed and methanol-treated fins were rehydrated from methanol to 1x PBS in a series of washes and then embedded into a 1.5% agarose/5% sucrose/1x PBS block. The block was equilibrated overnight in 30% sucrose. Embedded fins were mounted in onto a sectioning chuck with Tissue-Tek OCT, and transverse cryosections were taken using a Bright OTF5000 cryostat microtome. Sections were collected on Superfrost Plus slides (Fisher, 12-550-15) and air dried overnight at room temperature. Slides were stored at −20°C. Before use, slides were brought back to room temperature for at least 1 h. Sections were circled with a hydrophobic marking pen (ImmEdge Pen H-4000; PAP pen, VWR Laboratories) and blocked overnight at 4°C in an appropriate blocking solution. Sections stained with the commercial antibody against TMEM184A (1:100, Santa Cruz sc-163460) were blocked in 2% BSA/1x PBS. Sections stained with ZF-CTD (1:100) were blocked in 2% BSA/2x PBS. A slightly higher salt concentration (in the 2x PBS) was used for the ZF-CTD primary antibody to displace endogenous HS chains potentially bound to Tmem184a and make the antigen more accessible. Mouse anti-GFP (1:200) was added to all primary antibody solutions (prepared in respective blocking solutions), and sections were incubated with primary antibodies overnight at 4°C. Slides were washed in 1x or 2x PBS (twice for 5 min) and the blocking solution (once for 5 min) before being incubated with secondary antibodies for 30 min in 37°C. Secondary antibodies were prepared in the blocking solution. To detect mouse anti-GFP, Alexa 488 anti-mouse (1:200; 715-545-150) was used. To detect the commercial goat anti-TMEM184A primary antibody, Cy3 (705-165-147) or TRITC (705-025-147) anti-goat secondaries were used. To detect the ZF-CTD primary antibody, Cy3 (711-165-152) or Alexa 594 (711-585-152) anti-rabbit secondaries were used. Slides were washed again as before and mounted with 100% glycerol. Slides were stored at 4°C. To confirm specificity, ZF-CTD was pre-incubated with the blocking peptide and showed virtually no staining (data not shown). Sections were imaged on a Nikon C2+ confocal microscope with a 60x oil immersion lens.

### Percent similarity analysis and statistics

To account for variation between individuals and within one fin, regeneration lengths, and cell proliferation measurements are reported as the mean percent similarity between the injected side and the uninjected side as previously reported (Banerji et al., 2016). The percent similarity is calculated as ([measurement of the injected side/measurement of the uninjected side] × 100%). Values close to 100% indicate no effect of a MO or treatment; values <100% indicate that the measured parameter in the injected side is less than the uninjected side, and values above 100% indicate the injected side parameter is greater than the uninjected side. Graphs are shown as the mean % similarity ± SEM. Percent similarity comparisons between each experimental treatment vs. its corresponding control were analyzed using Student's *t*-test. Differences with *p* < 0.05 were considered significant. ^*^p < 0.05; ^**^p < 0.01; ^***^p < 0.001.

## Results

### Tmem184a is expressed in zebrafish vasculature and is required for normal regeneration

**Figure 1:**
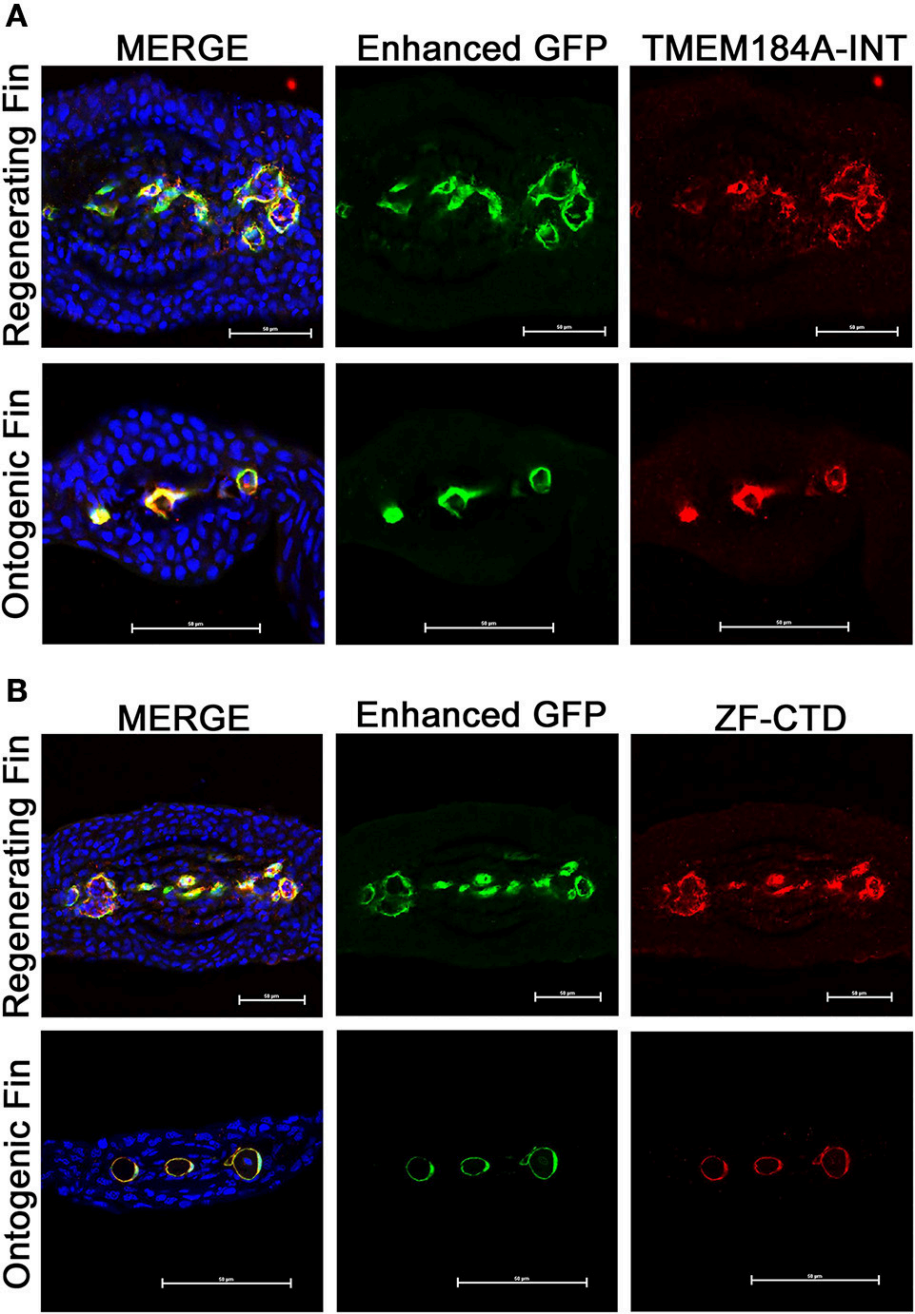
Ontogenic and regenerating zebrafish fins express Tmem184a in the vasculature. Representative confocal microscopy images of cryogenic transverse sections of regenerating and ontogenic *Tg(Fli1:EGFP)* fins. All sections were stained with a GFP antibody to enhance EC fluorescence/visualization along with DAPI to easily identify fin rays. **(A)** Sections were stained for Tmem184a using a commercial antibody. **(B)** Sections were stained for Tmem184a using a zebrafish-specific antibody (ZF-CTD). Each image shows one individual fin ray. The arrowhead points to a mature artery, and the arrows mark mature veins. Scale bars = 50 μm. DAPI staining is blue, EC staining is green, and Tmem184a staining is red for all images. Sections from more than 10 individual fish were immunostained and analyzed for each combination of antibodies.

We have recently reported the *in vitro* expression and function of TMEM184A in vascular cells. TMEM184A has yet to be studied in the vasculature *in vivo*. To first analyze whether zebrafish express Tmem184a, we immunostained transverse cryosections of ontogenic and regenerating *Tg(Fli1:EGFP)* fins. Sections were incubated with commercial primary antibodies against GFP (to optimize EC visualization) and TMEM184A (TMEM184A-INT). We observed remarkably specific expression of TMEM184A in ECs of both ontogenic and regenerating sections (Figure 1A). There was no staining in secondary antibody only controls (data not shown). To confirm staining specificity, we generated an antibody against the zebrafish Tmem184a protein. Immunostaining transverse sections with a zebrafish-specific antibody (ZF-CTD) showed similar specific staining of Tmem184a in the vasculature of ontogenic and regenerating fins (Figure 1B). Sections stained with the primary antibody pre-incubated with the blocking peptide showed no staining, confirming specificity (data not shown).

**Figure 2:**
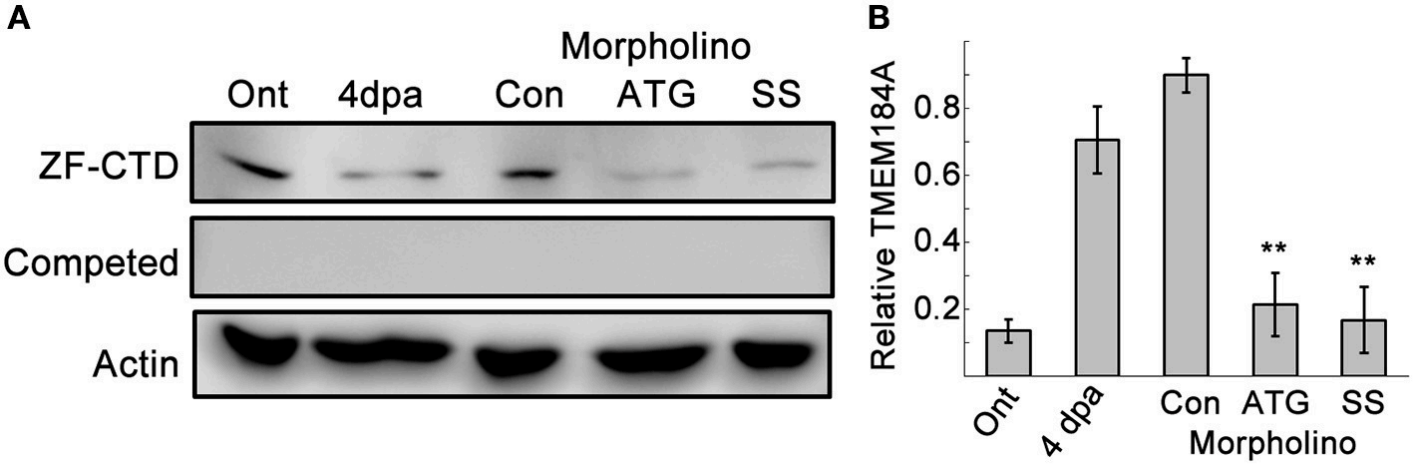
Two MOs against Tmem184a sufficiently knock down protein expression. **(A)** Validation of Tmem184a knockdown by western blotting of ontogenic and 4 dpa fin lysates with the ZF-CTD antibody. **(B)** Quantitation of Tmem184a band intensity/actin band intensity. Data are representative of more than three independent experiments with 8–12 fish injected per experiment. Graphs are shown as average ratio ± S.E.M. ^**^p < 0.01 compared to Con MO.

We expected Tmem184a to be essential for vascular function, and so we relied on MO-mediated knockdown to evaluate gene function during adult fin regeneration. We adhered to published guidelines for well-controlled MO-knockdown experiments including the use of two non-overlapping MOs compared to a control MO, confirmation of reduced target protein, and completion of at least three biological replicates when evaluating regeneration phenotypes (Eisen and Smith, 2008; Blum et al., 2015; see Methods for complete experimental procedures). Our Tmem184a targeting MOs included one ATG-blocker and one splice blocker compared to a non-targeting standard control MO. All MOs used in the study were tagged with a fluorophore (either fluorescein or lissamine rhodamine), permitting the confirmation of cellular uptake. Only fins positive for MOs at 24 h post electroporation were kept for further analyses. To demonstrate that both MOs target Tmem184a protein, MO-injected fin lysates were prepared as described in the methods and analyzed with ZF-CTD primary antibody (Figure 2A). Importantly, we found that both the ATG MO and SS MO reduced Tmem184a protein levels significantly as evidenced through immunoblotting (Figure 2). There were no bands detected with ZF-CTD pre-incubated with the blocking peptide, further demonstrating specificity (Figure 2A, Competed blot). Therefore, we concluded that both MOs were specific for the target.

**Figure 3:**
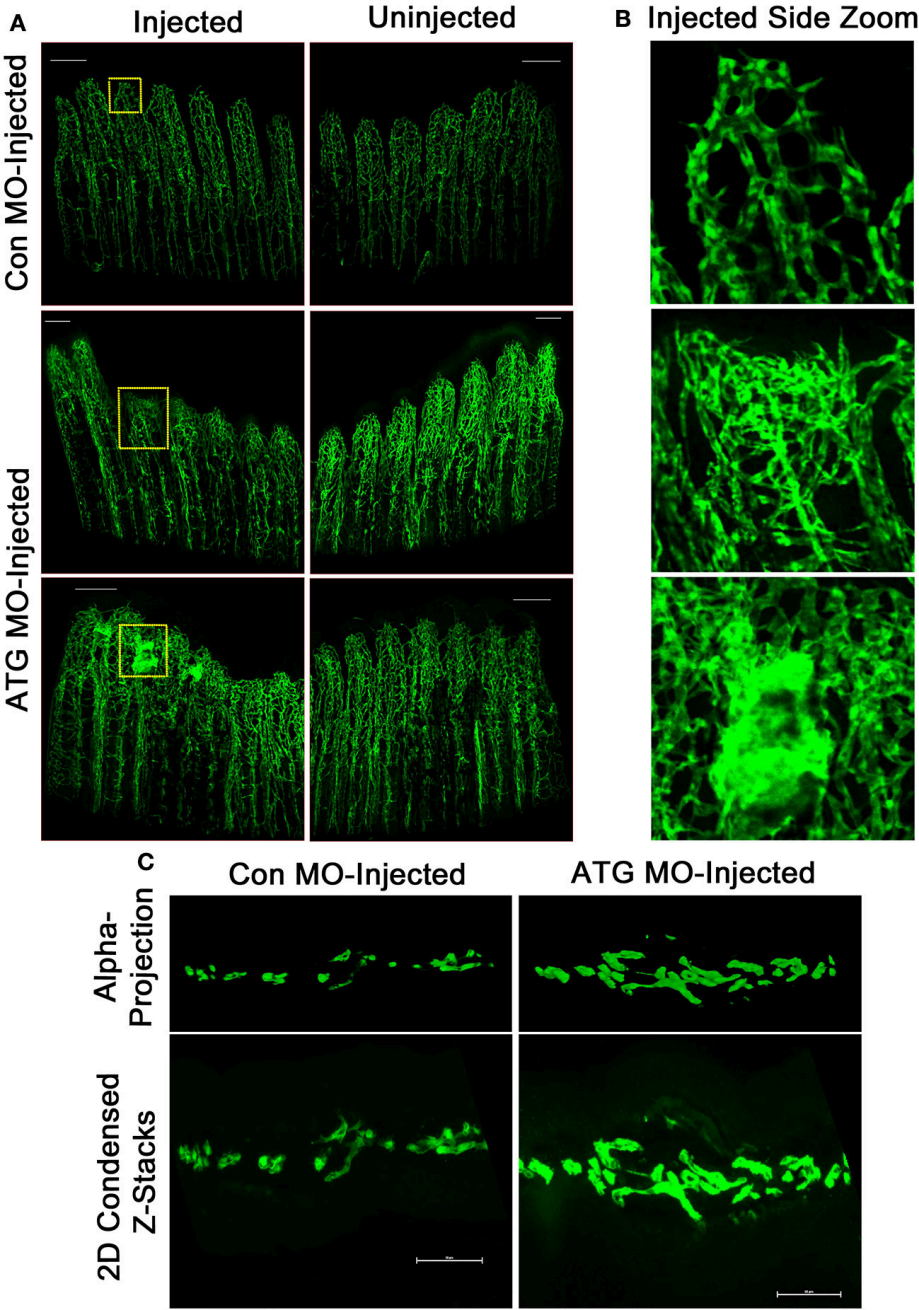
Tmem184a MOs-mediated knockdown results in aberrant vascular regeneration. Representative fluorescence microscopy images of fixed 4 dpa *Tg(Fli1:EGFP)* whole and cryosectioned fins. Each MO was injected into regenerating tissue of fin rays 1–6 at 3 dpa, and the other side was left uninjected. **(A)** There is no obvious difference in vascular phenotype between the side of a fin injected with Con MO vs. the uninjected side. Con MO *n* = 12. Injection with ATG MO results in two predominant phenotypes: impaired vascular outgrowth (top) and tangled regenerating vasculature (bottom). ATG MO *n* = 18. Scale bars = 200 μm. **(B)** 10x zoomed sections of injected fin shown in **(A)** allow easier discernment of regenerating vessels. **(C)** Example transverse maximum intensity projections (bottom) and alpha blended 3D projections (top) show the extent of vessel disorganization in an ATG MO-injected fin ray compared to one injected with Con MO. Scale bars = 50 μm.

We began by examining whether MO-mediated Tmem184a knockdown resulted in any abnormal vascular regeneration. To evaluate vasculature phenotypes, Con MO, ATG MO, or SS MO were injected at 3 dpa into the fin rays of one side of the fin, usually the dorsal side, and the other side was left uninjected. The entire fin was then electroporated. Fins were harvested 24 h later and fixed for analysis. Fins injected with Con MO did not exhibit any obvious vascular phenotypes different than the uninjected side (Figure 3A). Over additional days, the regenerating vasculature resolves from the vascular mesh surrounding the central artery and two more peripheral veins seen with significant diminution of the meshwork and retention of the three large vessels. Tmem184a knockdown resulted in two prominent phenotypes: significantly impaired vascular outgrowth (Figure 3A, middle panel) and a hypervascularized, “tangled” phenotype (Figure 3A, bottom panel). The regenerating vessels in the Tmem184a MO-injected fins were not organized into individual vessels as the control fin rays were (Figure 3B). All Tmem184a MO-injected fins had highly disorganized regenerating vasculature, and 50% of the individuals we observed had the tangled vascular phenotype. These two aberrant vascular phenotypes were apparent in both Tmem184a-targeting MO-injected fin rays, suggesting few off-target effects. MO-injected fins were cryosectioned to further observe the extent of vessel disorganization in individual fin rays (Figure 3C). Z-stacks were taken for each fin ray and condensed into a maximum intensity projection (bottom) to show overall vasculature fluorescence. Z-stacks were projected in 3D and alpha blended (top) to show the number and direction of regenerating vessels. The vessel disorganization in the ATG MO-injected fin rays was severe compared to Con MO-injected fin rays. The confocal images of the zoomed-in fin rays from the top-down and the cryosections illustrate why it was not possible to easily quantitate the extent of vessel disorganization observed in the fin rays where Tmem184a was knocked down.

**Figure 4:**
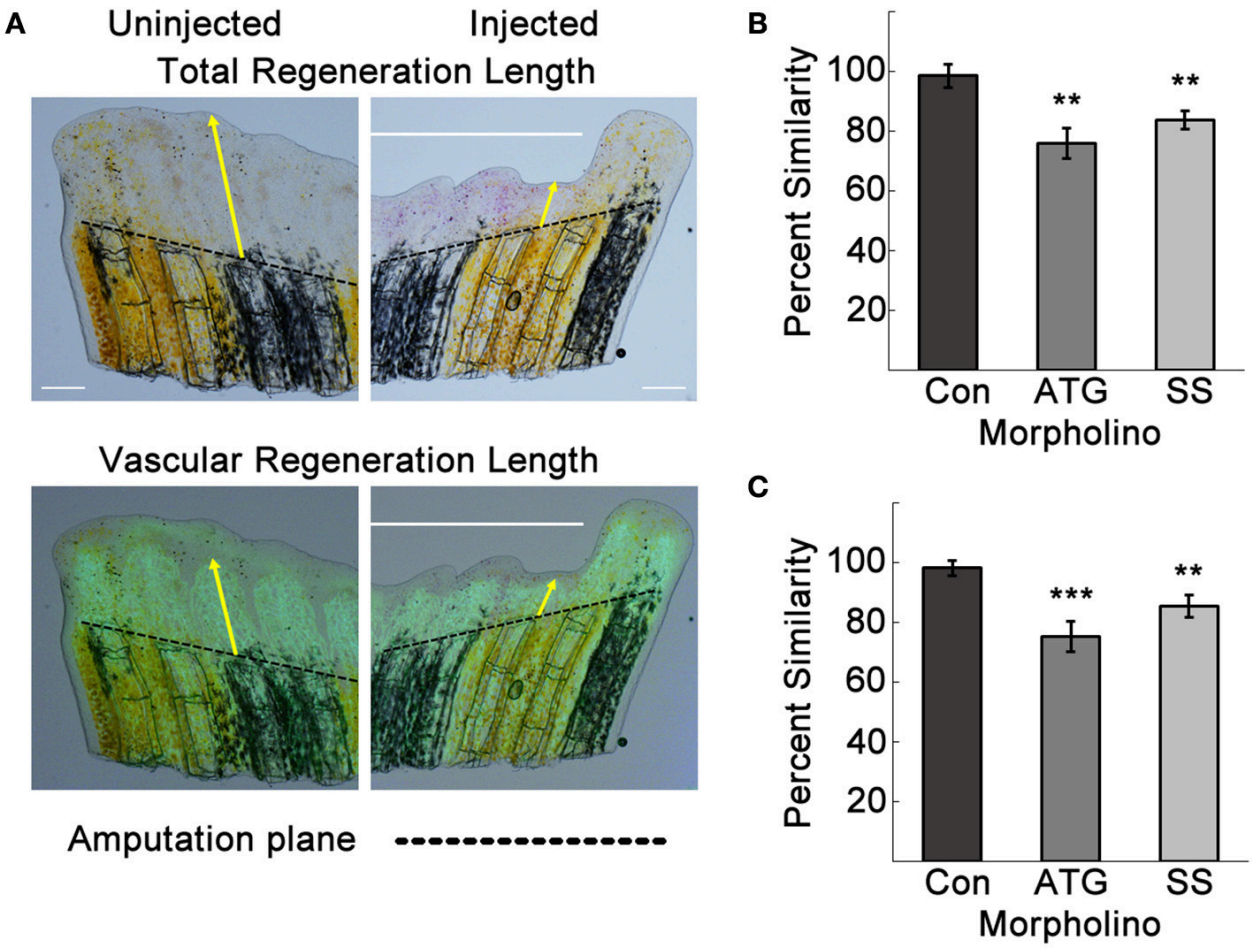
Tmem184a knockdown results in decreased total and vascular regeneration. Total and vascular regeneration of the third fin ray of the uninjected and injected sides of each fin were measured from the amputation plane (dotted line) to the tip of the fin and the tip of the regenerating vasculature. **(A)** Example brightfield image and superimposed vascular fluorescence image with yellow arrows pointing out total and vascular regeneration length measurements. **(B)** Quantitation of total regeneration length of the third fin ray of control, ATG, and SS MO-injected fins compared to the uninjected side. **(C)** Quantitation of vascular regeneration length as in **(B)**. Graphs are shown as mean percent similarity ± S.E.M. ^**^p < 0.01, ^***^p < 0.001 compared to control. Data are representative of more than three independent experiments with at least four fish per experimental group. Scale bars = 200 μm.

Improper vascular regeneration can prevent overall tissue regeneration. We quantified the total tissue and vascular regeneration lengths from the amputation planes of the third fin ray using ImageJ as previously described (Banerji et al., 2016; Figure 4A). Neither total nor vascular regeneration lengths were affected by the Con MO. However, in ATG MO and SS MO-injected sides, both total and vascular regeneration were decreased compared to the uninjected side. The percent similarities of the injected to uninjected sides for total and regenerated lengths were significantly different for both Tmem184a targeting MOs compared to the Con MO (Figures 4B,C).

### Tmem184a knockdown causes increased EC proliferation

**Figure 5:**
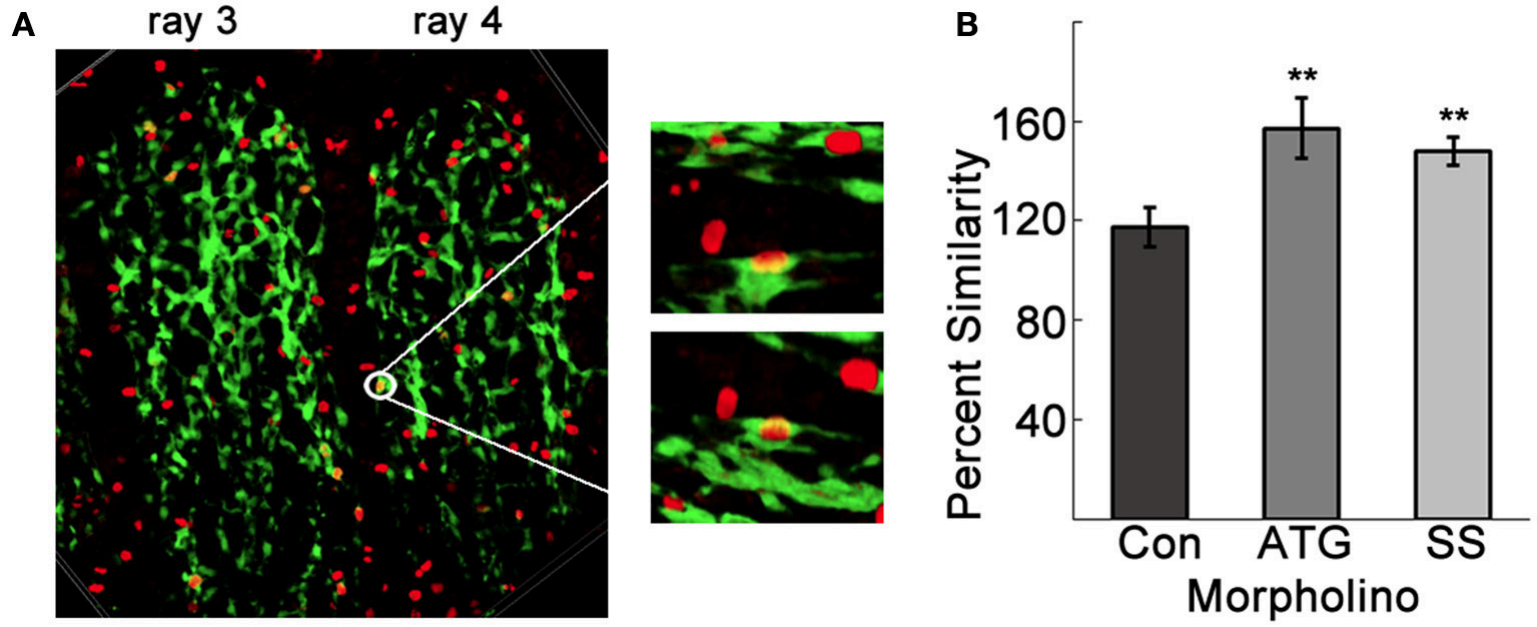
Tmem184a knockdown results in increased endothelial cell proliferation. **(A)** Example confocal image projecting the third and fourth 4 dpa fin rays. The white circle highlights a point where fluorescence from GFP (green, ECs) H3P (red, Cy3 secondary antibody) overlap and look yellow. Zooming in on this point and rotating in a few different directions confirms nuclear H3P in an EC. **(B)** Quantitation of H3P/Fli1 ECs in the third and fourth fin rays of 4 dpa standard control, ATG, and SS MO-injected fins compared to third and fourth fin rays of the uninjected side. Graphs are shown as mean percent similarity ± S.E.M. ^**^p < 0.01 compared to control. Data are representative of more than three independent experiments with at least four fish per experimental group.

The observed tangling of vessels in the Tmem184a knockdown fin rays suggested the possibility of an excess of ECs. We tested this by immunostaining for the mitosis marker histone 3 phosphate (H3P) in whole regenerating fins and quantified H3P in Fli1-positive ECs (Figure 5A). To observe only H3P-positive nuclei in ECs, we collected z stacks of the third and fourth fin rays of each side of MO-injected 4dpa fins. Using the show volume view function, the fin rays were projected with alpha blending. Any yellow nuclei above the amputation site were rotated in several directions to confirm a H3P/Fli1 positive EC. An example is shown in Figure 5A. There was no difference in the number of proliferating ECs between Con MO and uninjected fins. Not surprisingly, there was an increase in EC proliferation in ATG and SS MO-injected fin rays compared to the uninjected rays (Figure 5B). The percent similarity between the two sides was significantly different than the Con MO. The proliferation data in the Tmem184a knockdown fins nicely paralleled what we observed *in vitro* (Blaukovitch et al., 2010; Pugh et al., 2016). These data also suggest that Tmem184a is a negative regulator of EC proliferation.

### Heparin decreases vascular EC proliferation *In vivo*

**Figure 6:**
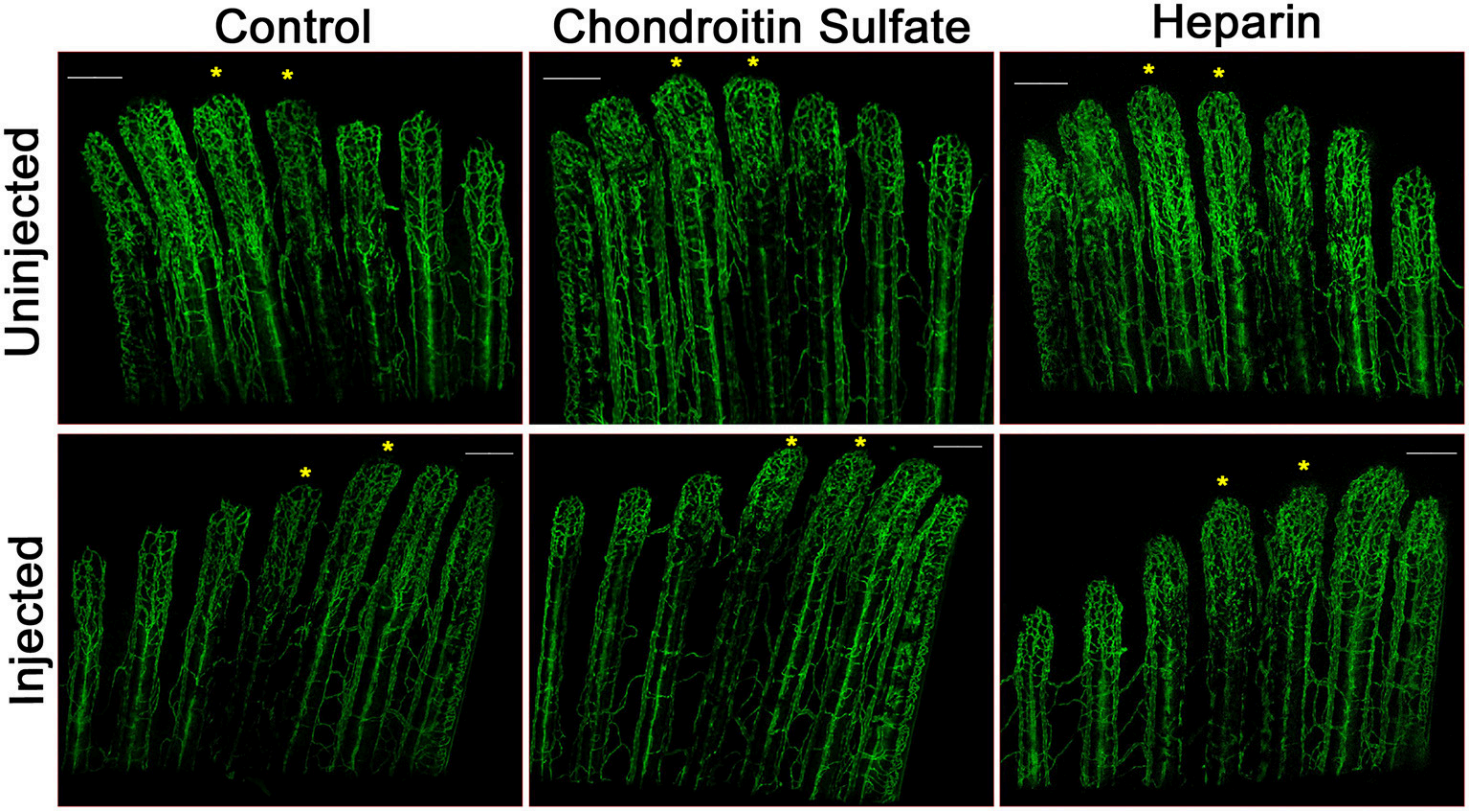
Heparin decreases vascular regeneration. Representative confocal microscopy images of 4 dpa *Tg(Fli1:EGFP)* fins. Fin rays 3 and 4 of one side of a fin (noted with asterisks) were injected at 3 dpa with either with phenol red (Control), chondroitin sulfate, or heparin, and the other side was left uninjected. A slight decrease in vascular outgrowth and a delay in central artery and vein regeneration are observed in fin rays injected with heparin. Images are representative of three independent experiments with at least three fish per experimental group. Scale bars = 200 μm.

**Figure 7:**
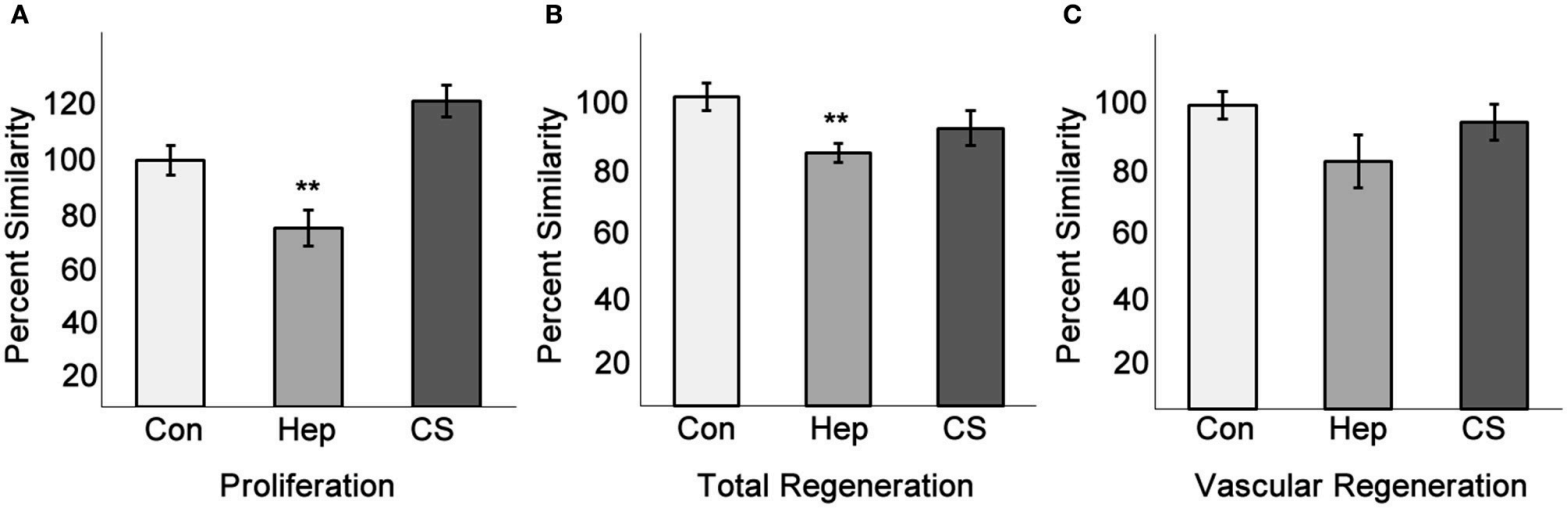
Heparin decreases endothelial cell proliferation and total fin regeneration. Fins injected with phenol red, heparin, or chondroitin sulfate were analyzed for EC proliferation, total regeneration length, and vascular regeneration length as in Figures 5, 6. **(A)** Quantitation of H3P/Fli1 ECs. **(B)** Quantitation of total regeneration length. **(C)** Quantitation of vascular regeneration length. Graphs are mean percent similarity ± S.E.M. ^**^p < 0.01 compared to control. Data are representative of three independent experiments with at least three fish per experimental group.

**Figure 8:**
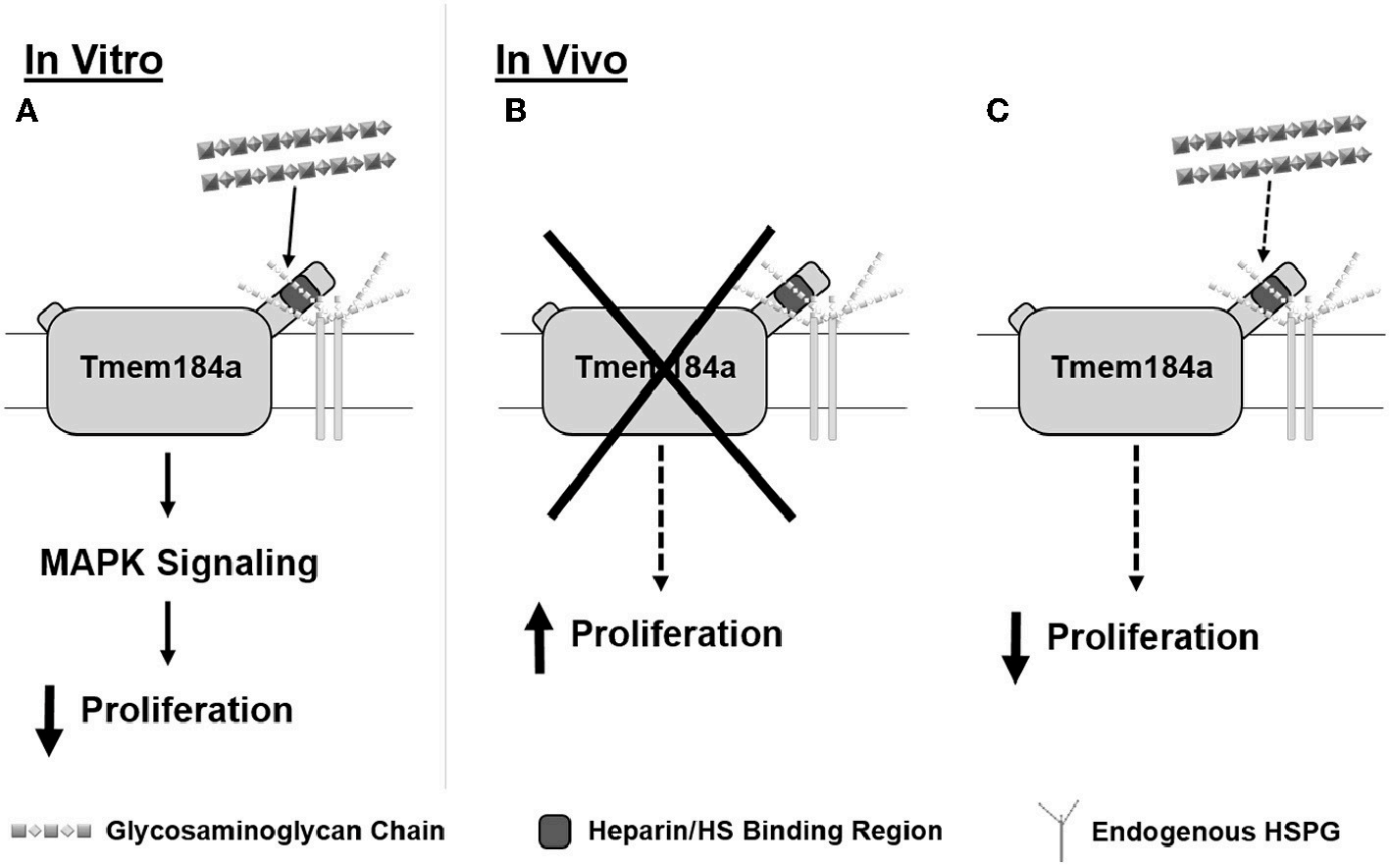
Schematic summarizing heparin/HS-dependent Tmem184a effects on vascular cell proliferation *in vitro* and *in vivo*. We have shown Tmem184a is expressed specifically on vascular cells. **(A)** *In vitro*, we have shown that exogenous heparin binds specifically to TMEM184A to induce MAPK signaling which decreases proliferation. **(B)** When Tmem184a is knocked down *in vivo*, cell proliferation increases. **(C)** Administration of excess heparin *in vivo* will also decrease cell proliferation, even though endogenous HS are present.

We have shown that exogenous heparin treatment decreases proliferation through Tmem184a in vascular cells (Gilotti et al., 2014; Pugh et al., 2016). We next wanted to see whether these effects on proliferation could be recapitulated *in vivo*. We injected heparin into the third and fourth fin rays of one side of 3 dpa fins and harvested at 4 dpa for phenotype analysis and the H3P assay. Chondroitin sulfate was used as a control for heparin due to its similar size and sulfation and does not mimic heparin effects. Phenol red alone (normally used to visualize the injection solution) was used as a negative control. There was a decrease in vascular regeneration in heparin-injected fins compared to controls (Figure 6). We also observed a delay in central artery and vein formation in heparin-injected fin rays compared to uninjected fin rays. H3P immunostaining indicated that there were significantly fewer proliferating ECs in fins injected with heparin compared to the uninjected side (Figure 7A), which matches what we have reported *in vitro*. There were no significant differences between the injected and uninjected sides of the phenol red or chondroitin sulfate controls. We also measured total and vascular regeneration lengths in these fins and found that both total and vascular regeneration were decreased in the heparin injected fin rays compared to the uninjected side. This decrease was only significant for total regeneration (Figures 7B,C). Thus, while the vascular outgrowth was not significantly hindered, the decrease in EC proliferation likely affected surrounding tissues and hindered normal total fin regeneration. These data further support the hypothesis that heparin specifically decreases vascular proliferation *in vivo* (Figure 8).

## Discussion

We have recently reported the *in vitro* expression and function of TMEM184A (Farwell et al., 2016; Pugh et al., 2016). Using human, bovine, and rat tissue culture systems, we have shown that TMEM184A is expressed in vascular ECs and SMCs. TMEM184A functions as a heparin receptor that is required to transduce specific and persistent anti-proliferative signaling. Exogenous heparin binds specifically to TMEM184A in a concentration-dependent manner to induce expression of DUSP1. DUSP1 inactivates the mitogen activated protein kinase (MAPK) ERK1/2 and its downstream target Elk-1 which ultimately results in decreased growth factor-induced proliferation (Figure 8A). To examine how the cellular roles of TMEM184A impact organismal function, it was necessary to move to an *in vivo* system. In the present study, we sought to examine the expression and function of TMEM184A *in vivo*. We chose the zebrafish regenerating caudal fin as an *in vivo* model for several important reasons.

TMEM184A is a highly-conserved protein in several species, including zebrafish, though its physiological function remains unknown. During our *in vitro* studies, we found TMEM184A expression to be significant in vascular cells; we needed to choose a model system in which we could test whether TMEM184A was expressed in the vasculature *in vivo* without performing invasive or complicated experiments. Genetic studies in the zebrafish fin have revealed conservation in the anatomy and function of the vasculature compared to other vertebrates (Gore et al., 2012). With the availability of several transgenic lines such as the *Tg(Fli1:EGFP)* line, visualization of the vasculature with microscopy is simple (Huang et al., 2003). Genetic manipulation is also straightforward, and standard validated protocols with appropriate controls are available. The ability of zebrafish fins to regenerate has also proven to be powerful for understanding the mechanisms of normal and pathological angiogenesis (Chávez et al., 2016). Genetic and toxicological screens for angiogenic therapies have been feasible, including high throughput tests for antibodies, natural products, and other chemical ligands targeting regulators of angiogenesis (Bayliss et al., 2006). Thus, the regenerating fin presented itself as a simple, yet versatile *in vivo* model system where we could elucidate the expression of Tmem184a and ask whether changes in proliferative signaling through Tmem184a would affect vascular regeneration specifically.

Here we report Tmem184a is specifically expressed in vascular ECs. Our results show that MO-mediated Tmem184a knockdown significantly increased EC proliferation, supporting our *in vitro* observations (Figure 8B). Exogenous heparin had the opposite effect on EC proliferation (Figure 8C). Additionally, total and vascular regeneration were decreased in both Tmem184a knockdown and heparin-injected fins. These effects of Tmem184a knockdown and heparin on vascular cell proliferation are remarkably similar to what we have observed *in vitro* (Pugh et al., 2016). We also expect that Tmem184a is expressed in zebrafish SMCs, and additional studies will be designed to address that question.

Proper angiogenesis requires the formation of a complex with VEGF isoforms and their receptors VEGFR1 and VEGFR2 (Flt1 and Flk1 in zebrafish), EC adherens junction proteins such as VE-cadherin, co-receptor Neuropilin-1, and certain HSPGs (Fuh et al., 2000; Bayliss et al., 2006; Whiteford et al., 2008). Members of this complex are conserved in the zebrafish and modulate angiogenesis in a similar fashion. Dynamic interactions between HS chains, VEGF isoforms, and VEGFRs in the complex activate signaling pathways that lead to changes in vessel permeability, cytoskeleton dynamics, and cell survival genes, which contribute to proper vessel sprouting (Lamalice et al., 2004; Xu et al., 2011; Ashina et al., 2015). HS sequesters VEGF through strong interactions to control binding to receptors. At the same time, HS chains on neighboring cells trap VEGFRs at the plasma membrane in part to regulate the concentration of available receptors at the membrane for precise growth factor binding (Jakobsson et al., 2006). During angiogenesis, HS chains direct VEGF binding to VEGFRs. VEGF binding to VEGFR2 leads to activation (phosphorylation) of the receptor which signals internalization. It has been well-documented that VEGFR2 and VE-cadherin internalization, trafficking, and recycling back to the plasma membrane are a crucial part of the regulation underlying angiogenesis and vessel outgrowth. There also is evidence showing that HS chains can be internalized along with at least VEGFR2 (Cheng et al., 2001; Zimmermann et al., 2005; Koch et al., 2011). Uptake and recycling of HS chains have been shown to stabilize proliferative signaling (Belting et al., 2002; Koch et al., 2011; Teran and Nugent, 2015). Furthermore, nuclear expression of HS has been observed (Cheng et al., 2001). In tumorigenic cells sensitive to growth factor signaling, internalized HS chains localized to the nucleus were thought to be involved in modulating transcription to decrease proliferation. It is possible that TMEM184A itself might endocytose with HS chains and other ligands that might be bound at the time to regulate proliferative signaling.

Prior to our *in vitro* work, there were only a few studies on TMEM184A. A couple studies done in Sertoli and exocrine cells showed TMEM184A involvement in vesicle trafficking (Best et al., 2008; Best and Adams, 2009). TMEM184A was shown to be required for secretory vesicles to be transported to their appropriate subcellular locations and recycled. Improper trafficking in TMEM184A knockdown cells resulted in severe cellular morphology changes. The authors also suggested that it was highly likely that TMEM184A might be working in a complex in order to have these effects. We have shown that heparin binds specifically to TMEM184A *in vitro*, likely at a putative binding site near the C terminus (Farwell et al., 2016, 2017; Pugh et al., 2016). We have also observed rapid heparin uptake into vesicles in vascular cells, and many of these vesicles also contain TMEM184A (Pugh et al., 2016; Farwell et al., 2017). *In vivo*, it is probable that HS chains are interacting with TMEM184A rather than free heparin, which is produced by mast cells as part of an immune response. Immunofluorescence staining of TMEM184A *in vitro* shows considerable expression in the perinuclear region (Best et al., 2008; Pugh et al., 2016). If a function of Tmem184a is to bind to HS chains to prolong sequestration of growth factors and receptors until proliferation is required, this would be consistent with the increase in EC proliferation we observed in Tmem184a knockdown fins.

Tmem184a knockdown resulted in a significant increase in EC proliferation, and heparin injection resulted in a decrease in EC proliferation, but both caused limited vascular regeneration and outgrowth. These results point to the idea that vessels must be organized in a way that ensures the new vessels would function properly (deliver nutrients) and promote tissue outgrowth. At the molecular level, there are several explanations for why these effects on proliferation would limit outgrowth including an imbalance of tip and stalk cells, effects on EC migration, displacement of HS chains by Tmem184a and/or heparin, and interactions with other heparin/HS binding proteins. During regeneration, ECs compete for the tip cell position which leads the sprouting vessel. HS chains are involved in tip/stalk cell maintenance through creating gradients of growth factors and chemokines to direct migrating ECs. Interactions between HS and Tmem184a required for maintaining such a gradient may have been disrupted in the Tmem184a knockdown fins. It is also possible that heparin is interacting through more than just Tmem184a. Migration of proliferating ECs has been shown to be controlled in response to VEGFR signaling, and altered Vegfr2 signaling in the Tmem184a MO and/or heparin-injected fins could have caused decreased migration. The regenerating caudal fin has limited supporting vascular cells (i.e., smooth muscle cells, mural cells, pericytes), and the literature suggests that these supporting cells are found in larger arterial vessels rather than the distal tips of newly sprouting vessels (Whitesell et al., 2014; Kametani et al., 2015). However, it is possible that injecting MOs or heparin into regenerating tissue would affect any supporting cells that would alter differentiation, migration, proliferation and survival of regenerating vasculature. Additional studies will be required to determine the mechanism(s) by which Tmem184a exerts its effects.

In conclusion, our data are the first to report an *in vivo* function for novel heparin receptor Tmem184a and suggest Tmem184a plays a regulatory role during angiogenesis. TMEM184A regulates angiogenesis by limiting EC proliferation and modulating outgrowth, likely through interactions with HSPGs. Few HSPGs and their binding partners have been identified and successfully targeted for angiogenic therapies. Further understanding of the role(s) Tmem184a plays in the vasculature will be critical for advancing our knowledge of mechanisms responsible for pathological angiogenesis and may also provide the opportunity for evaluating this novel receptor as a molecular target for angiogenic therapies.

## Author contributions

SF and KR carried out experimental work. SF, MI, and LL planned the study and wrote the manuscript. All authors analyzed data, reviewed and approved the final manuscript.

### Conflict of interest statement

The authors declare that the research was conducted in the absence of any commercial or financial relationships that could be construed as a potential conflict of interest.
